# Advances and challenges in modeling Charcot-Marie-Tooth type 2A using iPSC-derived models

**DOI:** 10.1016/j.stemcr.2025.102711

**Published:** 2025-11-13

**Authors:** Mafalda Rizzuti, Elisa Pagliari, Martina D’Agostino, Linda Ottoboni, Valeria Parente, Giacomo Pietro Comi, Stefania Corti, Federica Rizzo, Elena Abati

**Affiliations:** 1 Neurology Unit, Foundation IRCCS Ca’ Granda Ospedale Maggiore Policlinico, Milan, Italy; 2 Department of Pathophysiology and Transplantation, Dino Ferrari Center, Università degli Studi di Milano, Milan, Italy; 3 Neuromuscular and Rare Diseases Unit, Foundation IRCCS Ca’ Granda Ospedale Maggiore Policlinico, Milan, Italy

**Keywords:** CMT2A, MFN2, iPSCs, motor neurons, sensory neurons

## Abstract

Charcot-Marie-Tooth type 2A (CMT2A) is an inherited sensory-motor axonopathy caused by mutations in the *Mitofusin2* (*MFN2*) gene, coding for MFN2 protein. No curative treatment has been developed to date. The advent of induced pluripotent stem cell (iPSC) has provided unprecedented opportunities to understand complex neurological disorders. In CMT2A research, patient-specific iPSCs can be differentiated in motor and sensory neurons, thereby establishing reliable *in vitro* disease models. Here, we review current available iPSC-based models of CMT2A, focusing on pathogenetic insights derived from these studies and discussing challenges and potential of iPSC-derived models in elucidating disease mechanisms, providing innovative platforms for testing, and developing novel effective therapeutic strategies.

## Teaser

Abati and colleagues review iPSC-derived models of Charcot-Marie-Tooth type 2A (CMT2A), a hereditary neuropathy caused by MFN2 mutations. These patient-specific models successfully recapitulate disease hallmarks including mitochondrial dysfunction and impaired axonal transport, providing valuable platforms for understanding pathogenesis and testing therapeutic strategies like mitofusin agonists and HDAC6 inhibitors for this currently untreatable condition.

## Introduction

Charcot-Marie-Tooth disease (CMT) represents a clinically and genetically heterogeneous group of hereditary neuropathies, also defined as hereditary motor and sensory neuropathies (HMSNs), characterized by involvement of both motor neurons (MNs) and sensory neurons (SNs), which results in progressive weakness of the limbs, muscle atrophy, and loss of sensation (Harding and Thomas, 1980; Laurá et al., 2019; Morena et al., 2019; Pareyson and Marchesi, 2009; Pipis et al., 2019; Reilly et al., 2011). To date, there is no effective therapy for CMT patients, whose disability has a high impact on their quality of life. With a prevalence of 37/100,000 individuals, CMT is considered one of the most common neurological pathologies with a genetic component (Klein and Dyck, 2005; Laurá et al., 2019; Pisciotta et al., 2023; Pisciotta and Shy, 2023; Stuppia et al., 2015).

CMT disorders are divided into several types based on genetic and clinical differences, with type 1 (CMT1) and type 2 (CMT2) being the most common forms. CMT1 typically manifests in childhood or adolescence, while the onset of CMT2 is usually in adolescence or adulthood. Notably, CMT1 encloses multiple subtypes of demyelinating neuropathies, while the primary issue in CMT2 is axonopathy due to mutations in genes that affect the axons of peripheral nerves rather than the myelin sheath. On the basis of electrophysiological criteria, it is possible to distinguish these two CMT types, where demyelinating neuropathies present a nerve conduction speed lower than 38 m/s, while axonal neuropathies show a higher conduction speed (Harding and Thomas, 1980; Pareyson et al., 2017; Rossor et al., 2013; Saporta et al., 2011).

Among CMT2s, CMT type 2A (CMT2A) is the most frequent axonal dominant form, accounting for 20%–40% of CMT2s and ∼20% of all CMTs (Braathen, 2012; Fridman et al., 2015; Murphy et al., 2012). The typical clinical symptoms of CMT2A are represented by progressive weakness and atrophy of the distal muscles of the limbs, deformities of the feet, and loss of distal sensation. In some cases, motor difficulties can be very serious and require the use of walking aids. Alongside a more classic form, patients often experience the appearance of additional symptoms, such as optic atrophy, scoliosis, paresis of the vocal cords, hearing impairment, and, rarely, white matter alterations, epilepsy, or encephalopathy (Abati et al., 2022; Bombelli et al., 2014; Chung et al., 2006; Feely et al., 2011; Rouzier et al., 2012; Verhoeven et al., 2006; Züchner et al., 2004). Mutations in the *Mitofusin2* (*MFN2*) gene, which encodes for mitofusin 2, a key mitochondrial outer membrane protein involved in mitochondrial fusion and mitochondrial-endoplasmic reticulum (ER) tethering, are responsible for CMT2A (Cartoni and Martinou, 2009; Züchner et al., 2004). Over 100 different *MFN2* mutations have been identified across all protein domains, including the GTPase domains (i.e., R94Q, R94W, T105M, and H128Y) and coiled-coil domains (i.e., A418X, A707T, V705I, and R707W). These mutations contribute to disease pathogenesis through diverse and complex mechanisms. Indeed, many *MFN2* mutations act through a loss-of-function mechanism, impairing the role of the protein in mitochondrial fusion. However, some MFN2 mutations exhibit gain-of-function effects, where the mutant protein not only loses its normal function but also acquires new deleterious functions that interfere with other cellular processes on neurons. Furthermore, a dominant-negative effect can occur where a mutated MFN2 protein (whether due to loss or gain of function) interferes with the wild-type (WT) MFN2 protein, amplifying its negative impact on mitochondrial function. This ratio of WT to mutant MFN2 species plays a crucial role in disease severity (Chapman et al., 2013; El Fissi et al., 2018, 2020; Feely et al., 2011; Larrea et al., 2019). Mitochondrial DNA (mtDNA)-mediated inflammation has recently emerged as an additional pathogenic mechanism potentially involved in CMT2A, following the identification of the novel MFN2 Q367H variant in a patient with late-onset distal myopathy (Zaman et al., 2025).

Therefore, the pathological impact of MFN2 mutations cannot be fully explained by a single unifying mechanism, but it reflects a complex interplay of loss of function, gain of function, and possibly dominant-negative effects. This heterogeneity underscores the importance of characterizing the molecular behavior of individual MFN2 variants, as therapeutic strategies may need to be tailored accordingly, for example, restoring fusion in loss-of-function mutations or inhibiting toxic interactions in gain-of-function contexts (Amiott et al., 2008; Baloh et al., 2007; Chen et al., 2003; Detmer and Chan, 2007a, 2007b; Loiseau et al., 2007; Misko et al., 2010; Nicholson et al., 2008; Piscosquito et al., 2015; Polke et al., 2011; Tomaselli et al., 2016).

Given the considerable genetic and phenotypic variability observed in CMT2A, along with the involvement of both MNs and SNs in disease progression, developing reliable disease models remains both a critical and complex task for investigating pathogenesis and identifying effective therapeutic strategies. In this context, several models based on non-human cell types, such as mouse embryonic fibroblasts (MEFs) and murine dorsal root ganglia (DRG), as well as models using less disease-relevant cell types (e.g., fibroblasts) or knockout systems, have been employed. While these models can replicate certain disease-related features, they have reproduced heterogeneous results (Amiott et al., 2008; Baloh et al., 2007; Detmer and Chan, 2007a, 2007b; Loiseau et al., 2007). Recent advancements in induced pluripotent stem cell (iPSC) technology have significantly revolutionized the generation of disease-specific *in vitro* models. In the neurological field, iPSC-derived models have provided an innovative way to study complex disorders in a human context. As a matter of fact, patient-derived iPSCs can be differentiated into various disease-relevant cell types, providing the chance to model the disease *in vitro* and develop new therapeutic strategies, including personalized medicine approaches.

**Figure 1:**
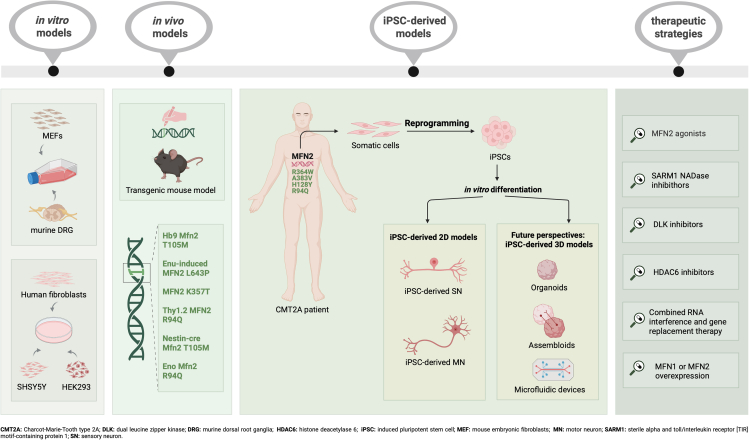
Experimental models and therapeutic strategies for CMT2A *In vitro*, *in vivo*, and iPSC-based models for disease modeling and translational applications. Created with BioRender.com.

Here, we revised the state of the art of the *in vitro* models developed for the CMT2A, based on iPSC-derived MNs and SNs (Table 1), and discussed the potential and limitations of iPSC-based models (Figure 1).

**Table 1:** Overview of current published iPSC-derived *in vitro* models of CMT2A

MFN2 mutation	Inheritance	Functional location	Control lines	iPSC-derived cell type	Differentiation protocol	Pathological remarks/Methodologies	Reference
R364W	AD	Linker region	Unaffected ctrl (*n* = 3)	MNs	Dual SMAD inhibition in adhesion culture followed by a caudal-ventral patterning step and magnetic bead sorting of L1CAM-positive cells (Burkhardt et al., 2013; Chambers et al., 2009)	- No alteration in mitochondrial number and size - Decrease of mitochondrial trafficking observed by live cell imaging - No alteration in neurofilament accumulation by immunofluorescent assay - Alterations in electrophysiological analysis (hyperexcitable state and changes in ion channel dynamics)	Saporta et al., 2015
A383V	AD	Linker region	Unaffected ctrl (*n* = 2)	MNs	Spontaneous neural induction and caudal-ventral patterning followed by centrifugation gradient enrichment (Hu and Zhang, 2009)	- No alteration in survival and morphometric parameters at immunofluorescence - Reduction in mitochondrial mass evaluated by RT-qPCR and WB - Alteration of mitochondrial positioning showed by immunofluorescence analysis - Decreased susceptibility to apoptosis and increased autophagic and mitophagic fluxes evaluated by RT-qPCR, WB, and immunofluorescence analysis	Rizzo et al., 2016
H128Y	AD	GTPase domain	Unaffected ctrl (*n* = 2)	MNs	Partial SMAD inhibition and caudal-ventral patterning through the EB-like aggregate method	- Abnormal mitochondrial morphology and fusion process at electron microscopy - Reduction in the percentage of moving mitochondria in TurboRFP-mito-transfected MNs shown by live cell imaging - Decrease in ATP levels in neurites determined by luminescent cell viability assay - Increased sensitivity to short-term exposure to anticancer drugs in terms of mitochondrial aggregation evaluated by immunofluorescence	Ohara et al., 2017
R94Q	AD	GTPase domain					
			Unaffected ctrl (*n* = 2) and CRISPR/Cas9 corrected MFN2^ISO^ iPSC line (*n* = 1)	MNs	Dual SMAD inhibition and caudal-ventral patterning through EBs (Guo et al., 2017; Maury et al., 2015)	- Significant decrease in neurite length and network (phase-contrast and immunofluorescence analysis) associated with hyper-connectivity measured with microelectrode array experiments - Alterations in mitochondrial morphology and axonal transport at live cell imaging - Oxidative phosphorylation defects observed by live-cell metabolic assay measuring oxygen consumption rate - Enrichment in PI3K-Akt, axon guidance, and respiratory chain pathways by bulk RNA sequencing	Van Lent et al., 2021
				SNs	Dual SMAD inhibition and rostral neural crest patterning through embryoid bodies (Chambers et al., 2012)	- Significant decrease in neurite length shown by phase-contrast imaging - Alterations in mitochondrial morphology and axonal transport by live imaging	

Summary of published iPSC-derived *in vitro* models of CMT2A, including neuronal subtypes, MFN2 mutations, and experimental approaches used to investigate disease mechanisms and potential therapies.

AD, autosomal dominant; ctrl, control individuals; MNs, motor neurons; SNs, sensory neurons; EBs, embryoid bodies.

## Modeling iPSC-derived motor and sensory neurons *in vitro*

Various approaches have been used to generate iPSC-derived MNs and SNs carrying CMT2A-associated MFN2 mutations. All begin with reprogramming patient cells into iPSCs and inducing neural fate, but they diverge in key technical details such as adherent versus aggregate cultures, neuronal enrichment methods, and MN or SN specification (Table 1).

The first patient-derived model was described by Saporta et al., in 2015. In this study, skin fibroblasts were reprogrammed into iPSCs and differentiated into spinal MNs using an adherent, stepwise protocol based on dual-SMAD inhibition, a widely used neural-induction strategy that suppresses non-neural fates and efficiently directs pluripotent stem cells toward a neural lineage. This approach enabled the generation of neural progenitors, which were subsequently specified into MNs. To further enrich the culture, magnetic-bead-based selection for L1CAM was applied, increasing the proportion of mature MNs while reducing the remaining neural progenitors (Saporta et al., 2015). Shortly thereafter, Rizzo et al. applied a multi-stage adherent protocol, originally developed for human pluripotent stem cells, which combined spontaneous neuralization with caudal-ventral specification over 5 weeks. Due to the limited accessibility of specific surface markers for MN isolation, culture enrichment was achieved through centrifugation gradient separation (Rizzo et al., 2016).

In contrast to adherent differentiation protocol, Ohara et al. induced MNs from iPSCs derived from peripheral blood cells using a free-floating protocol based on embryoid body (EB) formation coupled with SMAD inhibition and caudal-ventral patterning (Ohara et al., 2017).

Finally, Van Lent et al. generated both MNs and SNs from iPSCs through a dual-SMAD inhibition protocol: MNs were derived via a modified free-floating method, while SNs were generated based on a previously described protocol with additional compounds to promote neural crest specification and SN and maturation. Cytosine β-D-arabinofuranoside was added at the end of both differentiation protocols to enhance the purity of both MN and SN populations (Van Lent et al., 2021).

### MFN2^R364W^ iPSC-derived motor neurons

In 2015, for the first time Saporta and colleagues developed spinal cord MNs by reprogramming skin fibroblasts into iPSCs from a CMT2A patient harboring the p.R364W mutation in the *MFN2* gene (Saporta et al., 2015). iPSCs were differentiated into spinal cord MNs through an adherent, stepwise differentiation protocol (Burkhardt et al., 2013; Chambers et al., 2009) followed by magnetic-bead-based enrichment for L1CAM (Saporta et al., 2015). Analyses of MNs revealed normal morphology and an enrichment of markers associated with MN identity (i.e., ISLET1, HB9, and ChAT) and a reduction of markers associated with neural progenitors (i.e., PAX6 and OLIG2). While mitochondrial number and size were comparable in CMT2A versus control MNs, abnormal axonal mitochondrial trafficking has been observed in CMT2A MNs. Notably, mitochondrial movement along axons of CMT2A MNs was significantly slower in both anterograde and retrograde directions. Electrophysiological evaluation further revealed a hyperpolarized resting membrane potential, increased sodium current densities, and impaired channel inactivation. Overall, these findings suggest that CMT2A MNs display increased excitability and altered sodium and calcium channel function, features that may represent potential targets for therapeutic intervention.

### MFN2^A383V^ iPSC-derived motor neurons

In 2016, Rizzo and colleagues generated MNs from the skin-fibroblast-derived iPSCs of two CMT2A siblings, carrying the p.A383V *MFN2* mutation (Rizzo et al., 2016). Spinal MNs were differentiated from iPSCs using previously established multi-stage differentiation protocols that combine spontaneous neuralization with caudal-ventral specification followed by centrifugation gradient separation for MN enrichment (Hu and Zhang, 2009; Yu et al., 2007).

Affected MNs exhibited a global reduction in mitochondrial content, with reduced mtDNA content and mitochondrial protein expression impairment. A decrease in mitochondrial trafficking and perinuclear mitochondrial aggregation were also present, without significant differences in survival and axon elongation compared to healthy-subject-derived neurons, as already described in *ad hoc in vitro* models (Amiott et al., 2008; Detmer and Chan, 2007a, 2007b; Eura et al., 2003; Loiseau et al., 2007; Vielhaber et al., 2013) and in Saporta’s study (Saporta et al., 2015).

To provide a better description of CMT2A neuropathogenesis, for the first time in this study the authors performed RNA sequencing analysis of CMT2A MNs, identifying a downregulation of a p53-dependent apoptosis pathway in affected cells compared to MNs generated from healthy controls (reduced expression of pro-apoptotic caspase-8, BAX, and cleaved caspase-3 and increase of anti-apoptotic Bcl-2 expression). Furthermore, proteomic studies revealed an increased autophagy activation (overexpression of TFEB, BECN1, ATG5, and LC3B-II and reduction of autophagic substrate p62) as well as an upregulation of mitochondrial autophagic removal flux (upregulation of PINK1, PARK2, BNIP3, and BECN1s). Considering that cytosolic p53 has been associated with the suppression of autophagy and mitophagy in different models (Hoshino et al., 2012, 2013, 2014) and the increase of endogenous p53 levels in these CMT2A MNs showed the decrease of mitochondria clearance, the authors hypothesized that the increased mitophagy induced by p53 downregulation could be the leading pathogenesis disease mechanism for a massive reduction of mitochondria observed in these MNs expressing mutant MFN2 (Rizzo et al., 2016).

Taken together, these data suggest that changes in mitochondrial mass and distribution may play a role in disease pathogenesis by impairing mitochondrial transport or increasing mitochondrial turnover. Indeed, CMT2A-related MFN2 mutations may disrupt the interaction between MFN2 and motor proteins essential for axonal mitochondrial transport, resulting in increased pausing or stalling of transport (Baloh et al., 2007; Loiseau et al., 2007; Misko et al., 2010). Alternatively, mutant MFN2 may increase mitochondrial turnover via a gain-of-function mechanism that enhances interactions with proteins involved in mitophagy. These pathogenic events result in a decreased availability of mitochondria in neurons and disrupted transport into axonal compartments, as observed in patient-derived cells.

Overall, this study offers a comprehensive and detailed characterization of the pathological mechanisms underlying MN degeneration in CMT2A, establishing a valuable platform for the development and testing of new potential therapeutic strategies.

### MFN2^H128Y^ and MFN2^R94Q^ iPSC-derived motor neurons

In 2017, Ohara and colleagues generated MNs starting from peripheral blood mononuclear cells (PBMCs) or T-lymphocytes derived iPSCs from two patients carrying the p.R94Q and the p.H128Y MFN2 mutations (Ohara et al., 2017). Unlike previous protocols, the induction of MNs from iPSCs by SMAD inhibition and caudal-ventral patterning has been obtained using the embryoid body (EB)-like aggregate method. The authors observed a shorter mitochondrial length within neurites by electron microscopy, as well as the presence of abnormal mitochondria featuring loss of crista and apparent aggregations within neurites, suggesting an altered balance between mitochondrial fusion and fission. Furthermore, kymograph analysis by time-lapse experiments revealed that the percentage of moving mitochondria was significantly lower in CMT cells compared to controls, 40%–50% in controls versus 20% in CMT cells. The adenosine triphosphate (ATP) levels and the number of mitochondria in neurites resulted to be decreased in CMT neurons compared with controls, highlighting a mitochondrial dysfunction in affected neurites. Interestingly, treatment with the neurotoxic agents vincristine and paclitaxel induced mitochondrial aggregation in neurites both in CMT and control neurons with long-term exposure, although CMT neurons presented higher sensitivity to short-term exposure (Ohara et al., 2017). This is a notable finding, as it was speculated that anticancer drugs may induce neuropathy via mitochondrial changes, calcium signaling, or oxidative stress. In particular, vincristine was previously shown to cause impairment of mitochondrial fission, fusion, and mobility followed by axonal degeneration in DRG neurons from mice embryos (Cheng et al., 2024; Johns and Maragakis, 2022; Van Lent et al., 2021, 2024).

### MFN2^R94Q^ iPSC-derived motor and sensory neurons

In 2021, Van Lent and colleagues generated both MNs and SNs from a skin-fibroblast-derived MFN2^R94Q^ iPSC line (Van Lent et al., 2021). To generate spinal MNs, the authors used a slightly modified protocol originally described by Maury et al. (2015) to enhance the conversion of MN progenitors into post-mitotic spinal MNs as well as cell culture purity.

Alongside CMT2A cells, the authors also differentiated MNs starting from iPSCs derived from patients with other types of axonal CMT2, such as CMT2E (*NEFL*^P8R^), CMT2L (*HSPB8*^K141N^), and CMT2F (*HSPB1*^G84R^ and *HSPB1*^P182L^), to identify eventual common pathomechanisms that may open attractive avenues for therapy development. Since modifications in the neurite network have been described in iPSC-derived neuronal models as well as animal models of peripheral neuropathies (Perez-Siles et al., 2020; Ponomareva et al., 2016), the authors investigated this relevant phenotype in CMT2 iPSC-derived MNs by conducting neurophysiological analyses. Notably, patient MNs showed a significant decrease in neurite length associated with an increase in burst rate, suggesting hyper-connectivity. Moreover, a significant and progressive decline in both the speed and proportion of actively moving mitochondria and lysosomes was observed, supporting a generalized impairment of axonal transport, with trafficking of both organelles gradually deteriorating over time. Alterations in mitochondrial morphology (i.e., reduced mitochondrial elongation, increase in circularity, and decrease in aspect ratio) as well as a decrease in basal mitochondrial respiratory activity have been reported. Overall, defects in organelle trafficking and mitochondrial morphology were consistently observed across all CMT2 patient-derived lines; however, these impairments manifested earlier and more prominently in MN from CMT2A lines compared to other axonal CMT2 subtypes. Notably, the pathological rescue obtained in the isogenic MFN^ISO^ line generated by CRISPR/Cas9 technology supported the identification of true hallmarks of the disease, limiting the potential variability arising from genetic background differences. Moreover, bulk RNA sequencing performed on MNs showed that the axonal CMT samples clustered separately from the controls, identifying genes involved in respiratory chain, axon guidance, and the PI3K-Akt signaling pathways among the common differentially expressed ones, further supporting a link among CMT pathogenesis, mitochondria, and cell metabolism (Van Lent et al., 2021).

In addition to generating MNs, for the first time the authors differentiated the same CMT2 iPSC also into SNs, adapting a protocol reported in a previous study (Chambers et al., 2012).

In line with alteration observed in CMT2-derived MMs, CMT2A-iPSC-derived SNs showed a significant decrease in neurite length, mitochondrial dysfunction (i.e., reduction in the mitochondrial and lysosomal speeds and decrease in aspect ratio and mitochondrial shape). Collectively, these findings demonstrate a progressive mitochondrial dysfunction in both MN and SN across multiple CMT2 subtypes, highlighting the potential of targeting shared pathogenic mechanisms as a common strategy for developing effective therapeutic approaches for CMT2 disorders. Notably, the isogenic line showed a restoration of the disease-associated phenotype associated with the R94Q mutation in MFN2, providing evidence that phenotypic abnormalities identified in iPSC-derived MN and SN are reliable hallmarks of CMT2A.

## Advantages and limitations of iPSCs for CMT2A modeling

iPSC-derived models of CMT2A have elucidated pathogenic mechanisms that were not fully recapitulated by conventional animal models or primary cell cultures. Indeed, while mouse models often show milder phenotypes compared to human patients (Abati et al., 2024), iPSC-derived MNs consistently demonstrate robust mitochondrial dysfunction, altered trafficking, and electrophysiological abnormalities that mirror human disease characteristics. This species-specific advantage of human iPSC models is particularly important given that mitochondrial metabolism and axonal transport mechanisms can differ significantly between rodents and humans.

Different MFN2 mutations have been reported to result in distinct cellular phenotypes in iPSC-derived models, despite all causing CMT2A. The use of iPSC-derived cells offers a major advantage in this context, as it allows for patient-specific modeling of disease, enabling the study of mutation-specific effects in a human cellular environment. Convergent findings across all studies performed on iPSC include impaired mitochondrial axonal transport, altered mitochondrial morphology, and mitochondrial dysfunction, supporting these as core pathogenic mechanisms regardless of specific MFN2 mutation or differentiation protocol employed. However, mutation-specific differences are evident. The MFN2^R364W^ mutation (Saporta et al., 2015) preserved mitochondrial number while primarily affecting transport, whereas MFN2^A383V^ (Rizzo et al., 2016) caused global mitochondrial depletion, and MFN2^H128Y^/MFN2^R94Q^ (Ohara et al., 2017) showed prominent ultrastructural abnormalities. Notably, the electrophysiological abnormalities reported uniquely by Saporta et al. remain to be validated in other mutation contexts, highlighting a significant knowledge gap.

To date, studies investigating CMT2A using iPSCs (Ohara et al., 2017; Rizzo et al., 2016; Saporta et al., 2015; Van Lent et al., 2021) have primarily relied on control lines derived from unrelated healthy individuals. However, such controls often differ from patients in factors like age, sex, and genetic background, introducing potential variables that can obscure disease-specific molecular features. Notably, only Van Lent’s group (Van Lent et al., 2021) addressed this limitation by generating an isogenic control line in which the R94Q mutation in MFN2 was corrected using CRISPR/Cas9 genome editing technology. This strategy ensures that the control and mutant lines are genetically identical except for the disease-causing mutation, enabling more accurate attribution of observed phenotypes to the MFN2 variant. Isogenic controls are particularly valuable in autosomal dominant diseases like CMT2A, where they can help dissect how the ratio of WT to mutant MFN2 influences disease expression, providing a robust platform to determine whether correcting a single allele is sufficient to rescue the phenotype and offering insights into gene dosage effects. The use of isogenic lines also improves the identification of mutation-specific versus shared pathological mechanisms, thereby increasing the precision of potential therapeutic assessments. Critically, the use of isogenic controls represents a methodological advance that strengthens causal attribution of observed phenotypes to MFN2 mutations, while their demonstration of shared pathways across multiple CMT2 subtypes provides compelling evidence for common therapeutic targets.

Notably, iPSC-derived models present certain limitations that need to be considered when using them to study peripheral neuropathies. Indeed, while iPSC-derived MNs offer a promising tool for studying CMT, it has not yet been demonstrated that they can accurately mimic the progressive degeneration of long nerve fibers, which represents one of the key features of the disease. One of the most significant challenges in modeling axonal CMT2A is the failure to adequately reproduce the characteristic length-dependent dying-back pattern of axonal degeneration observed in patients. Indeed, in humans, the longest axons are preferentially affected, with degeneration typically beginning at distal terminals and progressing proximally. However, iPSC-derived neurons in traditional culture systems develop relatively short neurites (typically 100–500 μm) compared to the meter-long axons found in human peripheral nerves. This fundamental scale mismatch means that the bioenergetic stress and transport challenges faced by long axons *in vivo* cannot be fully replicated *in vitro*. In addition, the metabolic demands of long axons in peripheral nerves are substantially different from those of short neurites in culture. In human peripheral nerves, maintaining ionic gradients, supporting synaptic transmission, and powering axonal transport over long distances requires precisely regulated energy distribution along the entire length of the axon. iPSC-derived neurons may not experience the same metabolic stress, potentially masking subtle but critical defects in energy metabolism that become apparent only under the physiological demands of long-distance axonal transport. *In vivo*, peripheral axons are supported by complex interactions with Schwann cells, which provide metabolic support, growth factors, and structural guidance crucial for axonal energy metabolism. Most iPSC-derived CMT2A models lack these supporting cell types, potentially missing important aspects of disease pathogenesis related to axon-glial interactions. The absence of proper myelination in most culture systems further limits the physiological relevance of these models. Indeed, the three-dimensional architecture of peripheral nerves, including the endoneurial environment and blood-nerve barrier, cannot be replicated in conventional 2D culture systems. Also, peripheral axons *in vivo* experience mechanical forces including stretch, compression, and tension that may influence their vulnerability to degeneration. These physical stresses, which could exacerbate mitochondrial dysfunction in CMT2A axons, are absent in standard culture conditions.

In this regard, neuronal progenitor cells (NPCs) represent a particularly valuable model system for investigating the early emergence of mitochondrial dysfunction in peripheral neuropathy. These cells, being small and lacking the extensive neurite networks of mature neurons, do not rely heavily on mitochondrial mobility for their basic cellular functions, making them ideal for studying intrinsic metabolic defects before axonal transport complications arise. Importantly, recent work by Inak et al. demonstrated that in SURF1-related Leigh syndrome, mitochondrial defects observed in mature neurons were already detectable at the neuronal progenitor stage, revealing that metabolic dysfunction precedes morphological and functional abnormalities in differentiated cells (Inak et al., 2021). Their study showed that SURF1 NPCs failed to undergo the critical metabolic shift from glycolysis to oxidative phosphorylation, retaining a proliferative glycolytic state that prevented proper neuronal morphogenesis. This finding suggests that examining NPCs in CMT2A models could provide crucial insights into when and how mitochondrial defects first emerge during differentiation, potentially identifying therapeutic windows before irreversible axonal damage occurs. Furthermore, the relatively simple morphology of NPCs allows for more precise analysis of fundamental mitochondrial parameters without the confounding effects of complex axonal architecture.

Beyond their potential applications within the neurological field, iPSC-derived 2D models fail to capture the complex interactions between neuronal and glial cells, as well as the systemic interactions that occur *in vivo*. Furthermore, iPSC-derived cultures are inadequate to fully recapitulate the intricate process underlying neuromuscular junction (NMJ) formation, which is essential for MN function and communication with muscles, and it is compromised in CMT2A as a result of mitochondrial dysfunction. Current iPSC models typically assess isolated neurons rather than functional circuits, making it difficult to evaluate how mitochondrial dysfunction affects synaptic transmission and muscle innervation. Regarding SNs, so far only nociceptors have been derived, whereas axonal CMT primarily affects larger fibers (Van Lent et al., 2021). Nevertheless, the generation of both MNs and SNs from the same patient enables a direct comparison of how each cell type responds differently to disease or injury. Importantly, some phenotypes appeared earlier in MNs, providing insights into the temporal progression of the disease.

Finally, the establishment of standardized protocols for the generation of functional MNs and SNs, coupled with the adoption of consensus guidelines for phenotypic characterization, is critical for advancing the field and enabling the development of effective therapeutic strategies. Differences in cell source (skin fibroblasts or PBMCs), differentiation protocol, and enrichment technique can significantly affect results and may explain the phenotypic variability reported across studies. For instance, CMT2A typically manifests as a slowly progressive disorder over decades, yet iPSC-derived neurons are usually maintained in culture for a few weeks. This temporal compression makes it challenging to model the chronic, progressive nature of axonal degeneration. While acute mitochondrial dysfunction and transport defects can be observed relatively quickly in culture, the gradual axonal loss that characterizes the clinical phenotype remains difficult to capture. The question of whether short-term culture observations accurately predict long-term axonal viability remains unresolved. The implementation of harmonized methodologies and reproducible protocols for differentiation, culture conditions, and data analysis would greatly enhance the comparability and interpretability of results across studies.

Collectively, these studies establish iPSC-derived neurons as robust models for CMT2A research while emphasizing the need for standardized protocols and systematic comparative studies to distinguish mutation-specific from protocol-dependent phenotypic variations.

## Insights for therapeutic development

The development of therapeutic strategies for CMT2A has evolved from targeting individual pathways to recognize common mechanisms across different CMT2 subtypes, with some promising results in preclinical studies (Alberti et al., 2024; Rocha et al., 2018).

The diversity of therapeutic approaches explored for CMT2A reflects not only the complexity of its underlying pathogenesis but also the numerous potential points for clinical intervention, and while this variety is promising, it also introduces several strategic challenges that must be carefully considered. The timing of therapeutic intervention may play a critical role, as certain strategies might be most effective during periods of active axonal degeneration, whereas others could be more beneficial in presymptomatic individuals as a preventive measure. Beyond timing, the potential need for combination therapies adds complexity. Targeting multiple pathways might deliver synergistic benefits but also increases treatment complexity and the risk of drug interactions and off-targets effects. The heterogeneity of MFN2 mutations further heightens the possibility of unpredictable interactions and off-target effects, underscoring the need for rigorous preclinical validation and careful dose optimization as these approaches advance toward clinical application. Particularly, newer approaches such as gene therapy carry inherent challenges, including the risk of off-target effects and the need for efficient, safe delivery systems. These considerations are particularly relevant in CMT2A, where the widespread and predominantly peripheral distribution of affected motor and sensory neurons makes achieving adequate delivery to long axons especially difficult.

Patient stratification presents another challenge since the genetic and mechanistic diversity of MFN2 mutations may require highly personalized therapeutic strategies, complicating clinical development and regulatory requirements. Finally, the slow and variable progression of CMT2A further complicates trial design, demanding highly sensitive biomarkers and longer study durations to detect meaningful effects.

Notably, iPSC-based models have emerged as critical platforms not only for understanding disease pathophysiology but also for therapeutic screening and validation (Table 2).

**Table 2:** Overview of potential therapeutic applications for CMT2A

Therapeutic approach	Mechanism of action	Validation status	Advantages	Disadvantages	State of the art	References
Mitofusin agonists	Allosteric activation of MFN2, stabilizing fusion-permissive conformation and promoting mitochondrial dynamics	*In vitro:* iPSC-derived MNs from CMT2A patients *In vivo:* MFN2^T105M^ mouse model	- Disease-modifying strategy addressing root cause - Effective across different *MFN2* mutations - Drug-like properties with systemic administration - Improved pharmacokinetics in newer compounds	- Risk of over-fusion and mitochondrial clustering - Long-term safety profile unclear - Optimal dosing window narrow - Delivery challenges to peripheral nerves - Requires WT *MFN2*/*MFN1* allele	- MiM111 reversed neuromuscular dysfunction in mouse model with *MFN2*^T105M^ mutation - 4-hydroxy cyclohexyl analog showed enhanced cell permeability and restored mitochondrial motility in iPSC-derived MNs	Rocha et al., 2018 Franco et al., 2016 Franco et al., 2020 Franco et al., 2022 Dang et al., 2020
SARM1 inhibitors	Inhibition of SARM1 NADase activity, preventing NAD^+^ depletion and blocking axonal degeneration program	*In vitro:* iPSC-derived MNs from CMT2A patients *In vivo:* CMT2A rat model MFN2^H361Y^	- Addresses downstream axonal degeneration pathway - Broader applicability across multiple neurodegenerative diseases - Biomarkers available (cADPR levels) - Rescue metastable pool of damaged axons	- Risk of unintended consequences from blocking essential cellular process - Early intervention critical; limited efficacy after severe damage - Does not address upstream mitochondrial dysfunction - Potential effects on normal development and immune responses	- DSRM-3716 effectively prevented axonal degeneration in iPSC-derived MNs from CMT2A patients - SARM1 deletion in CMT2A rat model completely rescued axonal degeneration, NMJ defects, muscle atrophy, and functional impairment	Hughes et al., 2021 Sato-Yamada et al., 2022
HDAC6 inhibitors	Inhibition of HDAC6 restores α-tubulin acetylation and improves axonal transport	*In vitro*: iPSC-derived MNs from peripheral neuropathy patients *In vivo*: MFN2^R94Q^ knockin mice	- Consistent efficacy across multiple CMT2 subtypes - Relatively broad therapeutic window - Systemic administration possible - Advanced development pipeline (EKZ-102 approaching clinical trials) - Favorable preclinical safety profile	- Multiple cellular functions beyond α-tubulin deacetylation - Concerns about off-target effects during chronic treatment - Does not address underlying mitochondrial dysfunction - Incomplete rescue of disease phenotype	- HDAC6 inhibition rescued defective axonal mitochondrial movement in MNs derived from iPSCs - SW-100 ameliorated pathological phenotypes in CMT2A mouse model - EKZ-102 advancing toward first-in-human studies	Picci et al., 2020 Shen et al., 2021
DLK inhibitors	Inhibition of stress-responsive JNK signaling pathway, preventing c-Jun phosphorylation and downstream apoptotic signaling	*In vitro:* iPSC-derived MNs from CMT2 patients (MFN2^R94Q^)	- Broad neuroprotective effects across CMT2 subtypes - Small molecule druggability - Targets early stress signaling - Phase I clinical trials ongoing for ALS (NCT02655614)	- Long-term inhibition risks disrupting normal stress responses and adaptive mechanisms - Essential roles in neuronal development and plasticity - Potential systemic side effects - Addresses downstream consequences rather than root causes	- Increased mitochondrial aspect ratio and basal respiration in MFN2^R94Q^ MNs - Partial restoration of mitochondrial transport speed and percentage of moving mitochondria	Van Lent et al., 2021 Genentech, Inc., 2020
Gene therapy (RNAi + gene replacement)	Combined silencing of both mutant and WT MFN2 alleles via shRNA, with concurrent restoration of WT MFN2 protein through RNAi-resistant cDNA	*In vitro:* iPSC-derived MNs from CMT2A patients *In vivo:* MitoCharc1 CMT2A transgenic mice (AAV9-mediated ICV delivery)	- Most direct approach targeting genetic defect - Potential for one-time treatment with long-lasting effects - Tissue-specific targeting possible - Demonstrated efficacy in both cellular and animal models	- Autosomal dominant nature may require knockdown of both alleles - Complexity of combined RNAi approach increases off-target risks - High manufacturing costs and regulatory requirements - Delivery challenges to PNS - Potential overexpression toxicity	- Combined RNAi and gene replacement increased mitochondrial content, normalized mitochondrial distribution and mitophagy in CMT2A iPSC-derived MNs - *In vivo* validation in MitoCharc1 mice via AAV9-ICV delivery in newborns	Rizzo et al., 2023
MFN1 overexpression	Restoration of MFN1:MFN2 balance by increasing MFN1 levels to compensate for dysfunctional MFN2	*In vitro:* SHSY-5Y *In vivo:* MFN2^R94Q^ transgenic mice with PrP-MFN1 overexpression	- Can rescue multiple CMT2A phenotypes without targeting mutant protein - MFN1 compensates for MFN2 dysfunction - Well-tolerated in nervous system - Complete or near-complete rescue of locomotor and visual deficits in mice - Potentially applicable across different MFN2 mutations	- Requires gene therapy delivery - The unique role of MFN2 in ER-mitochondria tethering not fully replaced by MFN1 - Optimal expression levels need careful titration - Long-term consequences of altered MFN balance unknown	- MFN1 overexpression in nervous system of MFN2^R94Q^ mice rescued locomotor activity, sensorimotor coordination, and vision loss - Restored mitochondrial aggregation and axon degeneration at molecular level	Zhou et al., 2019 Stavropoulos et al., 2023

Summary of candidate therapeutic strategies evaluated in *in vitro* and *in vivo* models of CMT2A, highlighting advantages, disadvantages, and state of the art.

Abbreviations: DLK, dual leucine zipper kinase; HDAC6, histone deacetylase 6; ICV, intracerebroventricular; iPSC, induced pluripotent stem cell; JNK, c-Jun N-terminal kinase; MFN, mitofusin; MN, motor neuron; NMJ, neuromuscular junction; PrP, prion protein promoter; PNS, peripheral nervous system; RNAi, RNA interference; SARM1, sterile alpha and toll/interleukin receptor [TIR] motif-containing protein 1; SHSY-5Y, human-derived neuroblastoma cell line; WT, wild-type.

### Mitofusin agonists

Mitofusin agonists represent the most mechanistically direct approach to CMT2A therapy, as they address the fundamental defect in mitochondrial fusion caused by MFN2 mutations. Indeed, these compounds stabilize the conformation of MFN2, potentially bypassing the dysfunction caused by pathogenic mutations. The therapeutic potential of mitofusin agonists has been demonstrated across multiple CMT2A models regardless of specific MFN2 mutation type (Rocha et al., 2018). Rocha et al. showed that these compounds successfully reversed mitochondrial abnormalities in CMT2A iPSC-derived MNs, improving both mitochondrial membrane potential and axonal transport. Recent developments have improved pharmacokinetic properties, with 4-hydroxy cyclohexyl analogs showing enhanced cell permeability and sustained mitochondrial motility restoration (Dang et al., 2020). Franco et al. developed a subset of small molecule activators that stabilize the fusion-permissive conformation of MFN2 (Franco et al., 2016, 2020). More recently, the same group introduced a new class of mitofusin activators, including the compound MiM111, which showed efficacy in reverting mitochondrial stasis and fragmentation in patient-derived MNs from CMT2A patients carrying different MFN2 mutations (Franco et al., 2022). Despite promising preclinical results, several concerns limit the immediate clinical translation of mitofusin agonists. Indeed, the long-term safety profile of chronic MFN2 activation remains unclear, particularly given that excessive MFN2 activity can lead to aberrant mitochondrial clustering and functional impairment (Huang et al., 2007). Furthermore, the optimal dosing window appears narrow, requiring careful titration to achieve therapeutic benefit without toxicity. Finally, delivery to peripheral nerves, where the pathology primarily manifests, may require specialized formulations or delivery systems that have not yet been fully developed. While mechanistically attractive, mitofusin agonists face significant regulatory and manufacturing challenges. The need for chronic administration in a slowly progressive disease necessitates exceptionally robust safety data, and the heterogeneity of CMT2A mutations may require personalized dosing strategies.

### SARM1 inhibition

Inhibiting the SARM1 (sterile alpha and toll/interleukin receptor motif containing protein 1) pathway targets the final common mechanism of axonal degeneration, providing potential therapeutic benefits across multiple CMT2 subtypes. This approach is particularly attractive because it addresses the downstream consequence of mitochondrial dysfunction rather than the upstream cause. The therapeutic potential of SARM1 inhibition has been validated in multiple disease contexts. Hughes et al. developed small molecule SARM1 NADase inhibitors, including DSRM-3716, which effectively prevented axonal degeneration in iPSC-derived MNs from CMT2A patients even when administered after the onset of damage (Hughes et al., 2021). Remarkably, animal studies demonstrated that SARM1 deletion in a CMT2A rat model not only rescued axonal and synaptic deficits but also improved motor function (Sato-Yamada et al., 2022). The ability to restore a metastable pool of axons destined for degeneration suggests a potentially transformative therapeutic window. The primary concern with SARM1 inhibition is the risk of unintended consequences from blocking a fundamentally important cellular process. SARM1 plays roles in normal development and immune responses, raising questions about long-term systemic effects. Additionally, while SARM1 inhibition can halt degeneration, it may not restore function to already damaged axons. The approach also requires careful timing, as intervention may need to occur before irreversible structural damage occurs. SARM1 inhibitors benefit from prior clinical development in other neurodegenerative contexts, with compounds advancing to phase I trials for amyotrophic lateral sclerosis (ALS). This regulatory precedent may accelerate development for CMT2A applications.

### HDAC6 inhibition

Histone deacetylase 6 (HDAC6) inhibition addresses the transport dysfunction that characterizes CMT2A by restoring α-tubulin acetylation and correcting axonal transport defects. HDAC6 inhibition has shown consistent efficacy across multiple CMT models and related neurodegenerative diseases. The approach is particularly appealing because it targets the impaired axonal transport, a shared pathological mechanism that affects multiple CMT2 subtypes. Preclinical studies using compounds such as tubastatin A and SW-100 have demonstrated restoration of mitochondrial transport and improved neuronal survival (Picci et al., 2020). The therapeutic window appears relatively broad, and compounds can be administered systemically. HDAC6 has multiple cellular functions beyond α-tubulin deacetylation, raising concerns about off-target effects during chronic treatment. The enzyme plays roles in protein quality control, immune responses, and transcriptional regulation, potentially complicating long-term safety profiles. Additionally, while transport restoration is beneficial, HDAC6 inhibition may not address the underlying mitochondrial dysfunction that drives CMT2A pathogenesis. Notably, beyond its application in peripheral neuropathy, this approach has shown promising results in preclinical models of ALS, enhancing axonal transport, reducing protein aggregation, and improving neuronal survival (Del Rosso et al., 2022; Fazal et al., 2021; Phipps et al., 2024; Stoklund Dittlau et al., 2021; Vanderlinden et al., 2025). While clinical trials have not yet begun, the HDAC6 inhibitor EKZ-102 is advancing toward first-in-human studies. The relatively mature drug development pipeline for this class of compounds provides a clear path toward clinical application.

### DLK inhibition

Dual leucine zipper kinase (DLK) inhibitors target stress-activated pathways that become dysregulated in CMT2A due to mitochondrial dysfunction. DLK inhibition showed promising results in restoring mitochondrial morphology and activity in MFN2ˆR94Qˆ MNs (Van Lent et al., 2021). The approach benefits from clinical precedent, as DLK inhibitors have progressed to phase I trials for ALS (NCT02655614). The broad neuroprotective effects suggest potential benefits across multiple CMT2 subtypes. The therapeutic window for DLK inhibition appears narrow, given the essential roles of the protein in neuronal development and plasticity. Long-term inhibition risks disrupting normal stress responses and adaptive mechanisms. The approach also addresses downstream consequences rather than root causes of CMT2A pathology.

### Gene therapy

Gene therapy represents a rational approach for treating disease progression based on tackling the underlying genetic cause. In this context, Rizzo et al. developed an innovative combined gene therapy approach involving RNA interference (RNAi)-mediated knockdown of both mutant and WT MFN2, coupled with overexpression of a functional, RNAi-resistant MFN2 (Rizzo et al., 2023). This approach significantly rescued the CMT2A MN phenotype *in vitro* by stabilizing the disrupted axonal mitochondrial distribution and restoring impaired mitophagy. Molecular correction of MFN2 was also successfully validated *in vivo* in the MitoCharc1 transgenic CMT2A mouse model. This strategy is particularly attractive because it can theoretically restore normal MFN2 function regardless of the specific mutation. Since CMT2A appears to primarily result from a dominant-negative effect rather than a toxic gain of function, it is relevant to explore and deepen the efficacy of the overexpression of MFN2 or MFN1 alone by gene therapy, independent of knockdown. It has been reported that excessive overexpression of MFN2 was able to induce aberrant mitochondrial clustering, characterized by the accumulation of small, fragmented, and functionally impaired mitochondria, leading to the activation of apoptotic pathways (Huang et al., 2007). Beside this, MFN2 overexpression has been also explored as a viable approach to mitigate aging-related muscle atrophy with potential applications for multiple muscle disorders (Cefis et al., 2024). Within CMT2A context, it has been reported that MFN2 or MFN1 overexpression was able to mitigate mitochondrial fusion and transport defects induced by MFN2 mutants, both *in vitro* (Stavropoulos et al., 2023) and *in vivo* transgenic models, without side effects (Zhou et al., 2019). Notably, the phenotypic rescue due to the overexpression of the WT isoform of MFN1, may be applied to different CMT2A cases regardless of the MFN2 mutation type. These findings underscore the potential of MFN-based gene overexpression approaches as an effective strategy for modulating disease phenotypes in CMT2A, supporting further exploration in preclinical and clinical settings.

## Limitations and future perspectives

Beyond identifying promising therapeutic targets, the implementation of iPSC-based models for therapeutic screening requires the development of robust, scalable readout systems that can efficiently assess drug efficacy across multiple conditions and compounds. High-content imaging platforms have emerged as particularly valuable tools, enabling automated acquisition and analysis of multiple cellular parameters simultaneously, including mitochondrial morphology, axonal transport dynamics, and cell viability. These systems can be integrated with automated liquid handling for compound addition and media changes, allowing for the screening of large compound libraries with minimal manual intervention. Medium-throughput approaches often focus on disease-specific readouts such as mitochondrial membrane potential measurements, ATP production assays, and automated analysis of neurite outgrowth and branching patterns. The integration of multi-electrode arrays with iPSC-derived neuronal cultures provides functional readouts of network activity and can detect subtle changes in neuronal excitability that may precede morphological alterations. Additionally, the development of reporter cell lines expressing fluorescent markers for specific cellular processes, such as mitochondrial dynamics, autophagy, or stress responses, enables real-time monitoring of drug effects on disease-relevant pathways. The standardization of these readout systems across different iPSC lines and laboratories is crucial for generating reproducible results that can support regulatory approval and clinical translation of promising therapeutic candidates.

Recently, advanced patient-specific iPSC-based culture systems, such as microfluidic chips, organoids, and assembloids, have emerged as highly versatile and powerful platforms for disease modeling and preclinical research. These sophisticated systems offer the unprecedented opportunities to mimic complex tissue architectures and cellular interactions *in vitro*, offering a more biologically meaningful disease model. As a matter of fact, by closely reproducing the complex environment of human tissues, they may provide a valuable tool for both basic research and the development of personalized treatments that overcomes the limitations of traditional 2D models (Van Lent et al., 2024).

Particularly, organoids are 3D miniaturized self-organizing structures derived from iPSCs or embryonic stem cells (ESCs) able to recapitulate the biological architecture of an organ. The complexity of organoids can be further enhanced by generating assembloids, which integrate multiple organoid types to better replicate the interactions between different tissues, providing a more comprehensive model of organ development, disease progression, and potential therapeutic responses. The incorporation of a vascular system to facilitate nutrient and drug transport could further enhance the growth and overall function of organoids/assembloids (Rizzuti et al., 2024).

So far, iPSC-derived organoids have been developed only for CMT1A, a genetic condition associated with duplication of the *Peripheral myelin protein 22* (PMP22) gene, which results in dysmyelination within the peripheral nervous system (PNS). Van Lent’s group generated for the first time self-organizing organoids containing various cell types of the PNS, including myelinating Schwann cells. To date, patient-derived models to study myelination defects are lacking as the *in vitro* generation of human Schwann cells is very challenging. This self-organizing organoid was able to recapitulate the biologically meaningful features of the disease, providing an excellent model for studying demyelinating diseases and supporting the development of therapeutic approaches aimed at reducing PMP22 expression (Van Lent et al., 2023).

As of now, organoids specifically derived for CMT2A have not been widely reported or established. Notably, the generation of NMJ and PNS organoids may provide innovative models to study how MFN2 mutations impair mitochondrial dynamics, axonal transport, and neuronal survival. Within NMJ organoids, MN degeneration could be studied in a controlled microenvironment, making it easier to observe how mitochondrial dysfunction impacts NMJ development and function, impairing the neuromuscular transmission. Furthermore, in conditions affecting the PNS such as neuropathies, the function of the DRG can be impaired, leading to sensory deficits. In this context, the generation of PNS organoids would represent an innovative model to investigate the pathological mechanisms underlying sensorial dysfunction in CMT2A.

While 3D organoid systems offer promising solutions to the limitations of traditional 2D cultures, they present unique technical challenges that must be addressed to effectively model CMT2A pathophysiology. One of the primary obstacles is the inherent heterogeneity observed in organoid cultures, which can significantly impact reproducibility and data interpretation. To mitigate this variability, researchers have implemented standardized protocols with defined culture conditions, automated bioreactor systems, and rigorous quality control measures including morphological screening and molecular characterization through single-cell RNA sequencing. Bioengineering approaches such as microwell arrays and controlled scaffolds further help standardize organoid size and cellular organization.

Examining axonal phenotypes within 3D structures requires specialized approaches that differ substantially from conventional 2D analysis. Advanced imaging technologies, including light-sheet microscopy and two-photon imaging, enable deep tissue visualization of axonal dynamics and mitochondrial transport within intact organoids. Tissue-clearing techniques combined with confocal microscopy allow comprehensive analysis of axonal architecture throughout the entire organoid structure. The integration of microfluidic devices with 3D cultures represents a particularly promising approach, as these systems can separate neuronal soma and axonal compartments while maintaining the complex cellular interactions present in organoid cultures.

For CMT2A modeling specifically, these 3D approaches enable more physiologically relevant assessment of mitochondrial distribution along extended axonal projections and provide insights into how metabolic dysfunction propagates through neural networks. Multi-electrode arrays adapted for 3D cultures allow functional assessment of network connectivity, while live-cell imaging of fluorescent organelle markers enables real-time tracking of transport dynamics within the complex 3D environment.

Despite these advances, current 3D models still cannot fully recapitulate the extreme axonal lengths characteristic of human peripheral nerves or completely replicate the intricate myelination patterns critical for CMT2A pathogenesis. Future developments will likely focus on creating more sophisticated multi-cellular models incorporating neurons, Schwann cells, and supporting cell types, potentially combined with mechanical stimulation systems to better mimic the *in vivo* biomechanical environment of peripheral nerves.

Overall, the field is rapidly evolving toward more complex and physiologically relevant models. The development of NMJ organoids and peripheral nervous system organoids and the integration of vascular components would provide unprecedented opportunities to study CMT2A pathogenesis in a more holistic manner.

## Conclusions

Despite inherent limitations, iPSC-based models have significantly advanced the understanding of the molecular and cellular mechanisms underlying CMT2A. Patient-specific iPSC-derived models have successfully recapitulated hallmark disease features, including mitochondrial dysfunction, defective axonal transport, altered electrophysiological properties, and dysregulated autophagy and mitophagy. These models have not only provided critical insights into disease pathogenesis but have also emerged as powerful platforms for therapeutic discovery and preclinical evaluation. Promising strategies identified through iPSC-based studies include DLK and HDAC6 inhibition, mitofusin activation, and gene therapy, demonstrating the translational potential of these systems.

A significant challenge is the phenotypic variability observed among iPSC lines, which can impede reproducibility and cross-study comparisons. This variability arises from differences in donor cell populations, reprogramming techniques, clonal heterogeneity, and differentiation protocols (Juneja et al., 2019). The generation of isogenic control lines either by correcting pathogenic mutations in patient-derived iPSCs or by introducing mutations into healthy control lines represents a promising strategy to minimize genetic background effects. Notably, phenotypic diversity has important implications for therapeutic development, as it suggests that treatment approaches may need to be tailored to specific mutations or mutation classes. For instance, mutations that primarily affect mitochondrial fusion may be more amenable to mitofusin agonist therapy, while those that primarily disrupt axonal transport might benefit more from HDAC6 inhibition or cytoskeletal stabilization approaches.

Nevertheless, modeling axonal degeneration in CMT2A remains technically challenging, particularly in replicating the length-dependent vulnerability of peripheral MNs. *In vivo*, the longest axons are affected first and most severely. Standard iPSC-derived MN cultures, with significantly shorter axons, fail to fully reproduce this aspect of the disease. Microfluidic devices that spatially separate proximal and distal axonal compartments may address this issue, revealing key differences in mitochondrial distribution and morphology in affected MNs. Similarly, 3D cultures may allow for improved tracking of neurite outgrowth and axonal transport dynamics, although they still fall short of capturing the extreme axonal lengths observed in patients. Moreover, current iPSC models often lack the cellular complexity and microenvironmental context of the human nervous system. CMT2A pathogenesis likely involves intricate interactions between MNs, Schwann cells, glial populations, muscle cells, and vascular or immune components. To more accurately model these interactions, researchers are increasingly utilizing coculture systems to study neuromuscular junctions or myelination, as well as complex 3D structures such as organoids and assembloids (Van Lent et al., 2024). These multicellular models better approximate the architecture and function of the PNS and may offer improved platforms for studying disease mechanisms and testing therapeutic interventions.

Finally, while cellular therapies using neuronal or mesenchymal stem cells have been investigated in related neuropathies such as CMT1 (Leal et al., 2008), to date no studies have explored these approaches specifically in CMT2A. Therefore, although potentially beneficial, stem-cell-based therapies for CMT2A remain an unexplored avenue for future research.

Looking ahead, the integration of iPSC technology with cutting-edge genetic engineering, multi-omics profiling, and data analysis holds great promise for accelerating both the pathophysiology and therapeutic development. Integrating multi-omics approaches, such as transcriptomics, proteomics, and metabolomics, into iPSC-based models might offer a powerful means to unravel the complex molecular networks underlying the disease pathogenesis and to identify novel biomarkers and therapeutic targets with high precision. The implementation of precision medicine strategies, leveraging patient-specific iPSC models to inform individualized therapeutic interventions, holds significant promise for advancing personalized care for CMT2A.

## Acknowledgments

We thank Associazione Progetto Mitofusina 2 ONLUS and Associazione Amici del Centro Dino Ferrari for their support. We also thank the Italian Ministry of Education and Research – MUR (“Dipartimenti di Eccellenza” Program 2023–27 – Dept. of Pathophysiology and Transplantation, Università degli Studi di Milano). This work was promoted within the European Reference Network (ERN) for Rare Neuromuscular Diseases. Figures were created with BioRender.com.

Fundings: this study was funded by the 10.13039/501100003196Italian Ministry of Health grant GR-2018-12365358 to F.R. (2018–2021) and by the Telethon grant (GMR23T2153) and Ricerca Corrente 2026 MoH to S.C. The Department of Pathophysiology and Transplantation, University of Milan, is funded by the Italian Ministry of Education and Research (MUR): Dipartimenti di Eccellenza Program 2023 to 2027.

## Declaration of interests

The authors declare no competing interests.
